# Video-based artificial intelligence for automated neonatal respiratory monitoring

**DOI:** 10.1007/s00431-026-07267-w

**Published:** 2026-07-28

**Authors:** Simone Nascimento Santos Ribeiro, Aline Elvina Rodrigues Fernandes, Maria Eduarda Ribeiro Rocha Vargas, Raquel de Carvalho Velame, Marcos Aurélio Tavares Filho, Richardson Naves Leão, Helton Maia, Silvana Alves Pereira, Maria da Glória Rodrigues-Machado

**Affiliations:** 1 Post-Graduate Program in Health Sciences of Medical Sciences Faculty of Minas Gerais (FCMMG), Belo Horizonte, Minas Gerais Brazil; 2 Federal University of Rio Grande Do Norte (UFRN), Natal, Rio Grande do Norte Brazil

**Keywords:** Newborn, Artificial intelligence, Computer vision, Respiratory monitoring, Noninvasive monitoring

## Abstract

Neonatal respiratory monitoring is essential for the early detection of clinical alterations, especially in preterm newborns. Conventional methods are limited by subjectivity and the use of contact sensors. This experimental technological development study was conducted according to the Standards for Reporting Diagnostic Accuracy Studies (STARD) guidelines. A computer vision–based system was developed: a fine-tuned YOLO11 model segmented the thoracoabdominal region of interest, the respiratory signal was extracted by optical-flow analysis of thoracoabdominal motion, and the respiratory rate was estimated by detrending and automatic peak detection; pose-based movement detection restricted the estimation to periods when the infant was still. The study was approved under protocols No. 4,744,993, 4,699,000, and 3,232,698 and classified as Technology Readiness Level (TRL) 5–6. Twenty-three neonatal recordings were included, comprising 3387 manually annotated frames for segmentation-model training and evaluation. At the time of recording, the newborns were between 3 and 15 days of postnatal age, had a postmenstrual age between 37 and 40 weeks, and were breathing spontaneously in room air. The sample consisted of neonates with a gestational age of 33 ± 1.76 weeks and weight of 1741.69 ± 393.97 g. Apgar scores were 7 ± 1.0 and 8 ± 1.0 at the 1st and 5th minutes, respectively. Fifty percent were female, and 75% were delivered by cesarean section. The system demonstrated robust thoracoabdominal segmentation with mean average precision (mAP) > 94% and continuous pose tracking. Respiratory dynamics analysis enabled respiratory-rate estimation during stable periods, with consistent performance supporting technical feasibility. The mean absolute error (MAE) was 2.1 breaths per minute (bpm) compared with the simultaneous clinical reference assessment. *Conclusion*: The system demonstrated technical feasibility for automated thoracoabdominal segmentation and non-contact respiratory-rate estimation during periods of neonatal stability. Given the small, single-center sample and the inclusion of newborns breathing spontaneously in room air, prospective multicenter validation is required before clinical implementation.

**Table:** 

**What is Known:** •* Neonatal respiratory monitoring still relies mainly on contact sensors and subjective clinical assessment.* *• Artificial intelligence and computer vision have shown potential for noninvasive neonatal monitoring, but automated respiratory analysis systems remain limited.*	
**What is New:** *• An artificial intelligence–based video system integrating YOLO and respiratory-signal analysis enabled automated neonatal respiratory monitoring.* *• The system identified respiratory rate during periods of neonatal stability, supporting objective and noninvasive respiratory assessment.*

**What is Known:**

•* Neonatal respiratory monitoring still relies mainly on contact sensors and subjective clinical assessment.*

*• Artificial intelligence and computer vision have shown potential for noninvasive neonatal monitoring, but automated respiratory analysis systems remain limited.*

**What is New:**

*• An artificial intelligence–based video system integrating YOLO and respiratory-signal analysis enabled automated neonatal respiratory monitoring.*

*• The system identified respiratory rate during periods of neonatal stability, supporting objective and noninvasive respiratory assessment.*

## Introduction

Respiratory failure during the neonatal period remains one of the leading causes of neonatal morbidity and mortality, accounting for approximately 20–25% of deaths in this population [1, 2]. Early identification of respiratory alterations is essential to reduce complications and improve clinical outcomes [2]. Neonatal respiratory monitoring still relies predominantly on contact sensors, such as electrodes and thoracic belts, which present important limitations, including risk of skin injury, frequent displacement, and signal quality loss. In addition, clinical assessment based on observation shows high interobserver variability [3, 4]. In recent years, artificial intelligence (AI) has been widely applied in neonatology, including predictive models for mechanical ventilation [5, 6], classification of respiratory diseases [7], and clinical prognosis, such as bronchopulmonary dysplasia [8, 9]. These models have demonstrated high performance, with area under the curve (AUC) values > 0.90 in some scenarios [5, 6]. Simultaneously, noninvasive monitoring techniques have been developed, including video analysis, spectroscopy, respiratory sound analysis, and thermal sensors [10–12]. Among these approaches, computer vision stands out for enabling the extraction of physiological signals without physical contact. Recent studies have demonstrated that the analysis of body movement and respiratory signals from video can provide reliable estimates of respiratory rate [3, 12]. However, gaps still remain in the integration of these techniques with robust automated systems for continuous monitoring. In this context, the present study proposes the development of an artificial intelligence-based system for automated analysis of neonatal respiratory dynamics using video.

## Methods

This experimental technological development study was conducted according to the Standards for Reporting Diagnostic Accuracy Studies (STARD) guidelines [13]. The study was based on secondary analysis of neonatal video data previously collected in clinical studies conducted at a Brazilian public tertiary maternity hospital. The study protocols were approved by the Research Ethics Committee of the Universidade Federal do Rio Grande do Norte under approval numbers 4,744,993, 4,699,000, and 3,232,698, corresponding to CAAE certificates 24,822,119.8.0000.5568, 44,712,221.5.0000.5537, and 08711019.7.0000.5292. Written informed consent had been obtained from parents or legal guardians in the original studies.

### Setting and data acquisition

Videos were obtained in the clinical environment of a neonatal unit during routine clinical monitoring. Recordings were acquired using smartphone cameras in the visible-light spectrum, predominantly at a resolution of 1920 × 1080 pixels and a native frame rate of approximately 30 frames per second, ranging from 24 to 30 frames per second across devices. Newborns were positioned supine and wore only a diaper, allowing unobstructed visualization of the thoracic and abdominal regions. A standardized acquisition protocol was followed regarding infant positioning and thoracoabdominal visibility; the camera was positioned approximately perpendicular to the diaphragmatic line and approximately 30 cm from the newborn.

Recording duration was not fixed in advance and ranged from 2 to 5 min across newborns. For respiratory-signal analysis, continuous analyzable segments were selected during periods of behavioral quiescence, defined as the absence of gross body movements.

### Reference standard (ground truth)

The reference respiratory rate was determined by direct visual inspection of thoracoabdominal respiratory movements over a continuous 60-s period. The clinical assessment was performed simultaneously with video acquisition by a trained healthcare professional with extensive experience in neonatal care. The assessor responsible for the clinical respiratory-rate measurement was blinded to the estimates produced by the automated system. The clinical reference value was not used as an input to the algorithm and was used only for subsequent comparison with the automated respiratory-rate estimate.

### Data annotation

The thoracic and abdominal regions were manually annotated frame by frame by a trained evaluator according to standardized anatomical delimitation criteria. A second independent evaluator reviewed annotation consistency, and discrepancies were resolved by the principal investigator.

The 3387 manually annotated frames were used as frame-level ground truth for training and evaluating the thoracoabdominal segmentation model. These frames represented a sampled subset of the recordings and did not correspond to the temporal window used for respiratory-rate estimation. Respiratory-rate estimation was performed over the full analyzable interval of each included recording.

### System architecture

The system integrates several artificial intelligence modules. The thoracoabdominal region of interest is segmented by a YOLO11 nano model (Ultralytics) fine-tuned for this task, and anatomical landmarks are tracked by a YOLO11 pose model (medium variant, 17 keypoints). From the segmented region of interest, the respiratory signal is extracted and the respiratory rate is estimated as described below.

The respiratory signal was obtained as the mean vertical component of the dense optical flow within the thoracoabdominal region of interest:

$$s(t) = {\langle {v}_{y}(x, y, t)\rangle }_{ROI}$$

The signal *s*(*t*) was detrended and smoothed, and its respiratory peaks were detected. The respiratory rate was obtained from the number of detected peaks *N* over the analysis window of duration *T* (in seconds):

1 $$RR=60\cdot\frac NT\left(breaths\cdot min^{-1}\right)$$

The system also provides an alternative respiratory-rate estimator based on spatiotemporal filtering with fixed 3D convolutional kernels (temporal-difference, 3D Laplacian, and Gabor) followed by a Fast Fourier Transform (FFT) frequency analysis. This alternative is implemented in the system but is computationally more demanding, so the results reported in this study were obtained with the optical-flow and peak-detection pipeline described above.

### Processing and analysis

The thoracoabdominal movement time series extracted from each recording were analyzed to estimate the respiratory rate.

### System performance evaluation

System performance was evaluated using the mean average precision (mAP) and the Intersection over Union (IoU), which quantified the accuracy and the overlap of the detected anatomical regions, respectively, and the mean absolute error (MAE) and root mean square error (RMSE), which quantified the respiratory-rate error and its variability; agreement with the clinical assessment was analyzed through the correlation between measurements.

### Ethical aspects

The study was approved by the Research Ethics Committee of Universidade Federal do Rio Grande do Norte (UFRN) under protocols No. 4,744,993, 4,699,000, and 3,232,698, in accordance with the Declaration of Helsinki.

### Technological maturity

The system was classified as TRL 5–6 (Technology Readiness Level), indicating a functional prototype validated in a relevant environment, according to the European Commission [14].

### Eligibility criteria

Videos were eligible when they were obtained from newborns with a postmenstrual age between 37 and 40 weeks at the time of recording, with adequate visualization of the thoracic and abdominal regions and sufficient image quality for computational analysis. Only recordings obtained while the newborn was breathing spontaneously in room air were included.

Videos were excluded when they presented severe thoracoabdominal occlusion, such as thick blankets or clinical materials covering the torso; inadequate local lighting; an acquisition angle that prevented reliable visualization of the thoracoabdominal region; excessive non-respiratory movement, including sustained crying; or absence of an adequate clinical reference respiratory-rate measurement.

### Sample

The analyzed sample consisted of 23 (twenty-three) distinct neonatal recordings selected from an initial pool of 84 recordings obtained from 38 newborns. At the time of recording, the newborns were between 3 and 15 days of postnatal age, had a postmenstrual age between 37 and 40 weeks, were breathing spontaneously in room air, and were positioned supine with adequate visualization of the thoracic and abdominal regions.

## Results

A total of 84 recordings from 38 newborns were screened. After application of the eligibility and technical-quality criteria, 23 distinct neonatal recordings were included in the final analysis and 61 were excluded, primarily because the acquisition angle or recording conditions did not allow reliable thoracoabdominal respiratory analysis. The included recordings comprised 3,387 manually annotated frames used for training and evaluating the segmentation model.

**Fig. 1:**
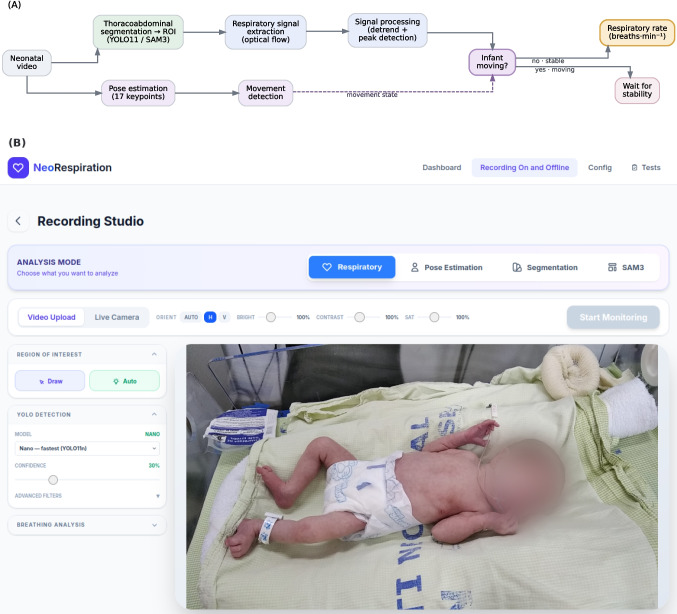
Overview of the video-based system. **A** Processing pipeline: from the neonatal video, thoracoabdominal segmentation defines the region of interest (a pre-processing step) and the respiratory signal is extracted by optical flow; the respiratory rate (the primary output) is obtained by detrending and peak detection. Pose estimation detects infant movement, which gates the computation: the respiratory rate is estimated while the infant is still, and the computation waits for stability whenever movement is detected, resuming once the infant is still again. **B** The NeoRespiration recording interface (newborn face de-identified) Source: real-time screenshots of the system’s output (authors’ own); newborn faces de-identified

The system’s processing pipeline and recording interface are shown in Fig. 1.

**Fig. 2:**
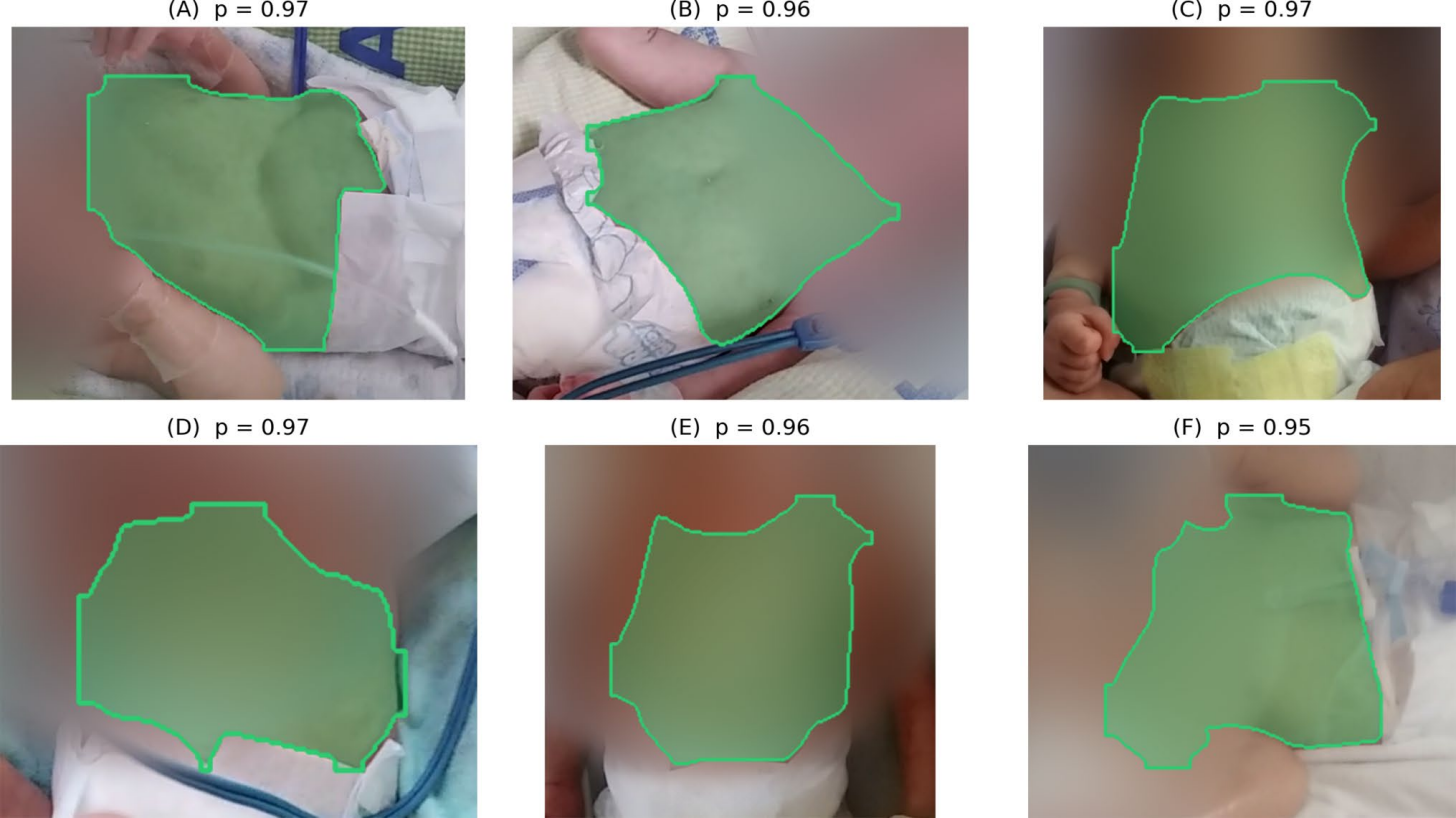
Automatic thorax–abdomen segmentation across six newborns. The green overlay and contour indicate the region predicted by the segmentation model (thorax and abdomen, excluding the diaper); p denotes the model confidence in each panel. Facial regions were blurred to preserve anonymity

The YOLO-based segmentation module demonstrated high accuracy in detecting the thoracic and abdominal regions, achieving mAP values greater than 94%, indicating robust and consistent performance in identifying the regions of interest throughout the analyzed frames. The model maintained satisfactory performance even under moderate variations in environmental lighting conditions and minor postural variations of the newborns, although slight performance reductions were observed in situations involving inadequate lighting, occlusions, or excessive neonatal movement. Representative thorax–abdomen segmentation results across different newborns are shown in Fig. 2.

Continuous tracking of anatomical regions, combined with pose detection, enabled the construction of stable respiratory movement time series.

Respiratory-rate estimation, obtained through optical-flow analysis with automatic peak detection, showed a mean absolute error of 2.1 breaths per minute compared with the simultaneous 60-s visual respiratory count performed by a blinded clinical assessor.

The analyzed recordings represented newborns with a mean gestational age at birth of 33.0 ± 1.76 weeks and a mean birth weight of 1741.69 ± 393.97 g. Mean Apgar scores were 7 ± 1.0 at 1 min and 8 ± 1.0 at 5 min. At the time of recording, the newborns were between 3 and 15 days of postnatal age and between 37 and 40 weeks of postmenstrual age. All newborns were breathing spontaneously in room air. Female newborns represented 50% of the analyzed sample, and 75% had been delivered by cesarean section.

**Fig. 3:**
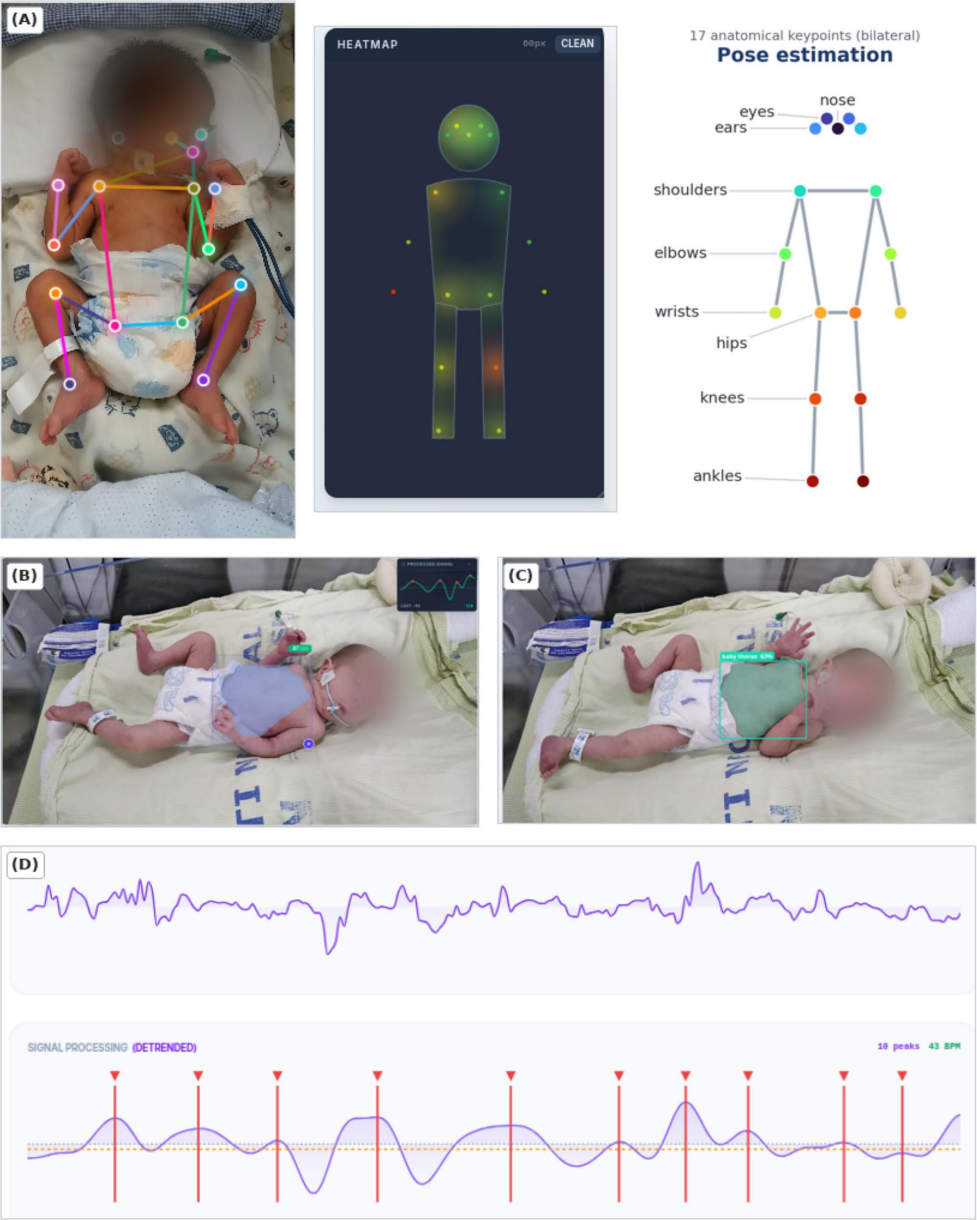
Features of the system in execution (newborn faces blurred for de-identification). **A** Pose estimation: anatomical keypoint tracking, a body-motion heatmap, and the 17-keypoint model, used to assess infant movement and activity. **B** Respiratory analysis: thoracoabdominal region-of-interest mask and the estimated respiratory rate (the primary output). **C** Zero-shot thoracoabdominal segmentation with SAM3, defining the region of interest as a pre-processing step for the respiratory analysis. **D** Respiratory signal processing: the extracted signal is detrended and the respiratory rate is obtained by peak detection

Figure 3 presents representative examples of the system’s features in execution, including thoracoabdominal segmentation, anatomical landmark tracking, and respiratory-rate estimation, illustrating the integrated analysis of neonatal respiratory dynamics.

Two thoracic-segmentation paths are provided depending on the available hardware. On GPU-equipped systems, SAM3 enables single-shot, zero-shot segmentation of the thoracic region directly from a text prompt, without any task-specific training, at the cost of higher computational demand. On CPU-only systems, a fine-tuned YOLO11 model provides efficient thoracoabdominal segmentation that runs in real time without a GPU, at the cost of requiring fine-tuning to adapt to new acquisition settings. The reported segmentation accuracy (mAP > 94%) refers to the fine-tuned YOLO model.

## Discussion

In this study, we developed and evaluated a video-based artificial intelligence system for automated analysis of neonatal respiratory dynamics. The main findings were that the system achieved a mean average precision greater than 94% for thoracoabdominal segmentation, enabled pose-based identification of periods of infant stability, and estimated respiratory rate with a mean absolute error of 2.1 breaths per minute compared with a simultaneous 60-s visual respiratory count performed by a blinded clinical assessor. Together, these findings demonstrate the technical feasibility of deriving respiratory information from visible-light neonatal videos through an integrated pipeline combining computer vision, motion analysis, and signal processing. Importantly, however, this performance was obtained in a small, single-center sample of clinically stable newborns breathing spontaneously in room air and should therefore be interpreted as evidence of technical feasibility rather than readiness for clinical implementation.

Accurate localization of the thoracoabdominal region was the first essential component of the processing pipeline. The fine-tuned YOLO-based segmentation model achieved mAP values greater than 94% and maintained satisfactory performance under moderate variations in lighting and minor postural changes. This finding is particularly relevant in neonatal video analysis because respiratory displacement is subtle and may be affected by changes in positioning, environmental conditions, occlusion, and spontaneous movement. Previous non-contact approaches based on video and computer vision have likewise demonstrated the feasibility of extracting physiological information from infant recordings [2, 3]. In the present study, however, reductions in performance under inadequate lighting, occlusion, and excessive movement also highlight that reliable anatomical localization remains dependent on acquisition quality and adequate visibility of the thoracoabdominal region.

Building on this anatomical localization, the system extracted thoracoabdominal motion through optical-flow analysis and generated respiratory movement time series during analyzable periods. Pose estimation served as a complementary component by identifying gross infant movement and gating respiratory-rate computation until stability was restored. This integration is relevant because spontaneous neonatal movements can introduce non-respiratory displacement into video-derived signals and compromise respiratory estimation. Rather than assuming that all recorded frames were equally suitable for analysis, the proposed pipeline restricted respiratory-rate estimation to periods of behavioral quiescence. This design choice may improve signal interpretability, but it also means that the current system should be understood as providing motion-gated respiratory monitoring during stable periods rather than uninterrupted continuous monitoring.

Based on the extracted respiratory signal, the system estimated respiratory rate with a mean absolute error of 2.1 breaths per minute relative to the simultaneous clinical reference. This result supports the feasibility of quantifying neonatal respiratory activity from visible thoracoabdominal displacement without physical contact. Previous studies have shown that respiratory information can be obtained through non-contact video-based approaches and through analysis of infant motion or physiological signals [3, 12]. The present findings extend this concept by integrating automated thoracoabdominal localization, optical flow–based signal extraction, peak detection, and pose-based movement gating within a single processing pipeline. Nevertheless, the observed error should be interpreted in the context of the limited sample size and the relatively homogeneous clinical condition of the included newborns.

The present approach also differs from other non-contact technologies explored for neonatal or infant monitoring. Thermal imaging, near-infrared spectroscopy, and acoustic analysis have demonstrated the potential to derive physiological or respiratory information without relying exclusively on conventional bedside sensors [10–12]. However, these methods differ in hardware requirements, signal source, and intended clinical application. By contrast, the proposed system derives its primary respiratory information from visible thoracoabdominal displacement recorded with smartphone cameras. This may offer practical advantages in terms of accessibility and scalability, while avoiding the need for specialized sensing hardware. At the same time, visible-light analysis remains sensitive to environmental conditions, body coverage, acquisition angle, and movement, as also reflected by the eligibility criteria and performance variations observed in the present study.

From a clinical perspective, these findings are relevant because neonatal respiratory assessment still depends on a combination of bedside observation and contact-based monitoring, both of which have recognized limitations [3, 4]. A contactless video–based approach could potentially complement existing monitoring strategies by providing objective respiratory-rate information during stable periods without adding sensors to the newborn’s skin. Such a role may be particularly relevant in neonatal care, where skin fragility, sensor displacement, and interobserver variability can affect monitoring and clinical assessment. However, the present study did not evaluate detection of clinical deterioration, reduction in adverse events, or improvement in clinical decision-making. Therefore, these potential applications should be considered future directions rather than demonstrated clinical benefits.

The potential value of automated respiratory information may become broader when considered alongside the expanding use of artificial intelligence in neonatal respiratory care. Previous studies have reported high predictive performance for outcomes such as the need for mechanical ventilation, extubation failure, and bronchopulmonary dysplasia using machine-learning models based predominantly on structured clinical and physiological data [5, 6, 8, 9, 15, 16]. In contrast, the present system focuses on dynamic visual information derived directly from neonatal thoracoabdominal motion. These approaches are therefore complementary rather than competing: predictive models estimate the probability of future clinical outcomes, whereas video-based respiratory analysis may provide repeated information about current respiratory dynamics. Future integration of these data sources could support more comprehensive models, but such integration was not evaluated in the present study.

This distinction is further supported by the broader literature on artificial intelligence applied to neonatal respiratory outcomes. Models have been developed for bronchopulmonary dysplasia prediction, risk stratification, respiratory infections, and prolonged respiratory-support requirements [17–21]. In parallel, computer-vision approaches have been used in neonatal intensive care settings for tasks such as automated detection of clinical interventions [22]. Taken together, these developments indicate a progressive expansion of AI from analysis of structured clinical variables toward interpretation of dynamic physiological and visual information. Within this context, the present study contributes a video-based approach focused specifically on thoracoabdominal localization and respiratory-rate estimation, while additional clinical validation remains necessary to establish its role alongside existing monitoring systems.

The clinical characteristics of the included newborns are central to interpretation of the observed performance. The analyzed recordings represented newborns with a mean gestational age at birth of 33.0 ± 1.76 weeks and a mean birth weight of 1741.69 ± 393.97 g; at recording, they were between 3 and 15 days of postnatal age and between 37 and 40 weeks of postmenstrual age. All were breathing spontaneously in room air. This relatively stable clinical condition may have favored thoracoabdominal visualization and reduced interference from respiratory interfaces, supplemental oxygen devices, and intensive bedside procedures. Consequently, the observed performance cannot be extrapolated to newborns with severe respiratory distress, marked instability, or those receiving noninvasive or invasive respiratory support. These populations may present additional challenges related to interfaces, tubing, positioning, greater work of breathing, and more frequent movement or clinical handling.

From a technological-development perspective, classification at Technology Readiness Level 5–6 indicates a functional prototype evaluated in a relevant environment [14]. The integration of thoracoabdominal segmentation, pose estimation, movement gating, respiratory-signal extraction, and respiratory-rate estimation within a single system represents an important step beyond isolated algorithmic testing. The availability of different segmentation pathways according to computational resources may also support future adaptation across heterogeneous settings. Nevertheless, technological maturity should not be equated with clinical validation, and the present findings should be interpreted within the methodological and clinical constraints of this initial evaluation.

Several limitations should therefore be considered. First, the study included a relatively small sample from a single center, limiting generalizability and preventing robust subgroup analyses. Second, the clinically homogeneous sample limits generalizability to newborns with severe respiratory distress, greater clinical instability, or receiving respiratory support. Third, recording duration was not fixed in advance, ranging from 2 to 5 min, which resulted in variability in the amount of analyzable respiratory information across newborns. Fourth, respiratory-rate estimation depended on periods of behavioral quiescence. When gross body movement occurred, computation was paused until stability was restored; consequently, performance during prolonged movement, crying, repositioning, or frequent clinical handling remains uncertain. Fifth, cesarean delivery accounted for 75% of the analyzed sample, reducing population diversity and potentially limiting the representativeness of the study population. Finally, the present study focused on respiratory-rate estimation and did not evaluate automated detection of clinical signs of respiratory distress, such as retractions or nasal flaring, nor its relationship with established severity scores such as the Silverman–Andersen score [23].

These limitations directly define the next steps for development**.** Future studies should prioritize prospective multicenter validation in larger and clinically heterogeneous populations, including newborns receiving supplemental oxygen, noninvasive respiratory support, and invasive mechanical ventilation. Evaluation under different lighting conditions, camera devices, acquisition angles, and levels of spontaneous movement will also be necessary to determine robustness in real-world neonatal environments. In addition, future extensions could investigate automated detection of thoracoabdominal asynchrony, paradoxical motion, intercostal and subdiaphragmatic retractions, supraclavicular retractions, and nasal flaring. Integration with electronic health records and hospital monitoring platforms may ultimately broaden the clinical utility of the system, but such applications will require dedicated validation of performance, interoperability, and clinical impact.

## Conclusion

The video-based system demonstrated technical feasibility for automated thoracoabdominal segmentation and respiratory-rate estimation during periods of neonatal stability. The low error observed compared with the clinical reference supports further development of the approach. However, prospective multicenter studies including newborns with different levels of respiratory support and clinical severity are required before clinical implementation.

## Data Availability

The datasets generated and/or analyzed during the current study consist of neonatal videos used for respiratory dynamics analysis. Due to the sensitive nature of these data and the potential for indirect participant identification, the datasets are not publicly available. Data were anonymized and stored in a secure environment with restricted access in accordance with ethical and regulatory requirements. Data may be available from the corresponding author upon reasonable request and subject to ethical approval.

## Declarations

### AI use policy

Generative artificial intelligence tools were used to assist with language editing and the preparation of this manuscript; the figures consist of real system outputs and author-prepared diagrams, and all scientific content, data, analyses, and results are the authors’ own and were reviewed and verified by the authors.

## Abbreviations

AI: Artificial intelligence
FFT: Fast Fourier Transform
IoU: Intersection over Union
MAE: Mean absolute error
NICU: Neonatal intensive care unit
RMSE: Root mean square error
RR: Respiratory rate
STARD: Standards for Reporting Diagnostic Accuracy Studies
TRL: Technology readiness level
VL: Visible light spectrum

## References

[1] Bjorland PA, Øymar K, Ersdal HL, Rettedal SI (2019) Incidence of newborn resuscitative interventions at birth and short-term outcomes: a regional population-based study. BMJ Paediatr Open 3(1):e000592. 10.1136/bmjpo-2019-000592 31909225 10.1136/bmjpo-2019-000592 PMC6936999

[2] Du YC, Chen PF, Ciou WS, Lin TW, Hsu TC (2024) An IoT-based contactless neonatal respiratory monitoring system for neonatal care assistance in postpartum center. Int Things (The Netherlands)28*:*101371. 10.1016/j.iot.2024.101371

[3] Khanam FT, Perera AG, Al-Naji A, Gibson K, Chahl J (2021) Non-contact automatic vital signs monitoring of infants in a neonatal intensive care unit based on neural networks. J Imaging 7(8):122. 10.3390/jimaging7080122 34460758 10.3390/jimaging7080122 PMC8404938

[4] Krupa AJD, Chauhan B, Azam SKS, Anand SA, Bereznychenko V, R N, Dhanalakshmi S (2025) Automated hypoxia and apnea identification for neonates via enhanced respiratory signal modeling with deep learning. Sci Rep 15(1):40898. 10.1038/s41598-025-24783-1 41257997 10.1038/s41598-025-24783-1 PMC12630711

[5] Im JE, Park S, Kim YJ, Yoon SA, Lee JH (2023) Predicting the need for intubation within 3 h in the neonatal intensive care unit using a multimodal deep neural network. Sci Rep 13(1):6213. 10.1038/s41598-023-33353-2 37069174 10.1038/s41598-023-33353-2 PMC10106895

[6] Kim Y, Kim H, Choi J et al (2023) Early prediction of need for invasive mechanical ventilation in the neonatal intensive care unit using artificial intelligence and electronic health records: a clinical study. BMC Pediatr 23:525. 10.1186/s12887-023-04350-1 37872515 10.1186/s12887-023-04350-1 PMC10591351

[7] Cho HW, Jung S, Park KH, Choi JW, Heo JS, Kim J, Yun H, Yu D, Son J, Choi BM (2025) Deep-learning-based multi-class classification for neonatal respiratory diseases on chest radiographs in neonatal intensive care units. Neonatology 122:446–454 40049153 10.1159/000545107

[8] Leigh RM, Pham A, Rao SS, Vora FM, Hou G, Kent C, Rodriguez A, Narang A, Tan JBC, Chou FS (2022) Machine learning for prediction of bronchopulmonary dysplasia-free survival among very preterm infants. BMC Pediatr 22(1):542. 10.1186/s12887-022-03602-w 36100848 10.1186/s12887-022-03602-w PMC9469562

[9] Choi HJ, Lee G, Shin SH, Lee SM, Lee HC, Sohn JA, Lee JA, Kim HS (2025) Development and external validation of a machine learning model to predict bronchopulmonary dysplasia using dynamic factors. Sci Rep 15(1):13620. 10.1038/s41598-025-98087-9 40253571 10.1038/s41598-025-98087-9 PMC12009281

[10] Hakimi N, Arasteh E, Zahn M, Horschig JM, Colier WNJM, Dudink J, Alderliesten T (2024) Near-infrared spectroscopy for neonatal sleep classification. Sensors (Basel) 24(21):7004. 10.3390/s24217004 39517901 10.3390/s24217004 PMC11548375

[11] Grooby E, He J, Kiewsky J, Fattahi D, Zhou L, King A, Ramanathan A, Malhotra A, Dumont GA, Marzbanrad F (2021) Neonatal heart and lung sound quality assessment for robust heart and breathing rate estimation for telehealth applications. IEEE J Biomed Health Inform 25(12):4255–4266. 10.1109/JBHI.2020.3047602 33370240 10.1109/JBHI.2020.3047602

[12] Lorato I, Stuijk S, Meftah M, Kommers D, Andriessen P, van Pul C, de Haan G (2021) Automatic separation of respiratory flow from motion in thermal videos for infant apnea detection. Sensors (Basel) 21(18):6306. 10.3390/s21186306 34577513 10.3390/s21186306 PMC8472592

[13] Bossuyt PM, Reitsma JB, Bruns DE, Gatsonis CA, Glasziou PP, Irwig L, Lijmer JG, Moher D, Rennie D, de Vet HC, Kressel HY, Rifai N, Golub RM, Altman DG, Hooft L, Korevaar DA, Cohen JF; STARD Group (2015) STARD 2015: an updated list of essential items for reporting diagnostic accuracy studies. BMJ 351:h5527. 10.1136/bmj.h5527. ID: 26511519 10.1136/bmj.h5527 PMC4623764 26511519

[14] European Commission (2014) Horizon 2020 Work Programme 2014–2015: General Annexes. Annex G: Technology Readiness Levels (TRL). European Commission, Brussels

[15] Tao Y, Ding X, Guo WL (2024) Using machine-learning models to predict extubation failure in neonates with bronchopulmonary dysplasia. BMC Pulm Med 24(1):308. 10.1186/s12890-024-03133-3 38956528 10.1186/s12890-024-03133-3 PMC11218173

[16] Gates Q, Ehwerhemuepha L, Janardhan S, Joshi R, Mikhael M (2025) Early prediction of mechanical ventilation needs in very preterm neonates using machine learning. Pediatr Pulmonol 60(7):e71195. 10.1002/ppul.71195 40728020 10.1002/ppul.71195 PMC12305751

[17] Verder H, Heiring C, Ramanathan R, Scoutaris N, Verder P, Jessen TE, Höskuldsson A, Bender L, Dahl M, Eschen C, Fenger-Grøn J, Reinholdt J, Smedegaard H, Schousboe P (2021) Bronchopulmonary dysplasia predicted at birth by artificial intelligence. Acta Paediatr 110(2):503–509. 10.1111/apa.15438 32569404 10.1111/apa.15438 PMC7891330

[18] Patel M, Sandhu J, Chou FS (2022) Developing a machine learning-based tool to extend the usability of the NICHD BPD outcome estimator to the Asian population. PLoS ONE 17(9):e0272709. 10.1371/journal.pone.0272709 36112600 10.1371/journal.pone.0272709 PMC9480997

[19] Torchin H, Dhiman P, Ancel PY, Durrmeyer X, Jarreau PH, Nuytten A, Truffert P, Zeitlin J, Collins GS (2026) Early prediction of bronchopulmonary dysplasia: comparison of modelling methods, development and validation studies. Pediatr Res 99(1):88–95. 10.1038/s41390-025-04170-2 40506584 10.1038/s41390-025-04170-2

[20] Kassaw A, Bekele G, Kassaw AK et al (2024) Prediction of acute respiratory infections using machine learning techniques in Amhara Region, Ethiopia. Sci Rep 14:27968. 10.1038/s41598-024-76847-3 39543232 10.1038/s41598-024-76847-3 PMC11564824

[21] Leigh BC, Liedl LM, Amsbaugh AL, Carey WA (2025) Spectral analysis of gastric aspirates obtained shortly after birth predicts the need for prolonged respiratory support in neonates in a development cohort. Front Pediatr 13:1686794. 10.3389/fped.2025.1686794 41458086 10.3389/fped.2025.1686794 PMC12738924

[22] Hajj-Ali Z, Dosso YS, Greenwood K, Harrold J, Green JR (2024) Depth-based intervention detection in the neonatal intensive care unit using vision transformers. Sensors (Basel) 24(23):7753. 10.3390/s24237753 39686290 10.3390/s24237753 PMC11644908

[23] Hedstrom AB, Gove NE, Mayock DE, Batra M (2018) Performance of the Silverman Andersen Respiratory Severity Score in predicting PCO2 and respiratory support in newborns: a prospective cohort study. J Perinatol 38(5):505–511. 10.1038/s41372-018-0049-3 29426853 10.1038/s41372-018-0049-3 PMC5998375

